# Tone of the Brain Improved by Disease

**Published:** 1849-03

**Authors:** 


### TONE OF THE BRAIN IMPROVED BY DISEASE.

Instances of this are not rare in the annals of medicine. The case of Dr. Priestley's son has often been quoted. Of a feeble mind by nature, he fell from a window and fractured his skull, and from that time exhibited powers of intellect decidedly improved. Dr. Sewall reported a similar case some years ago. A young man of naturally very limited intelligence lost a portion of his brain by a pistol shot, and after his recovery his friends were surprised to find him vastly improved in the activity and strength of his mind. He lived two years afterwards. Professor Dudley has been in the habit of mentioning in his lectures an analogous case. A young man received a blow on the front of his head by which the skull was fractured and he lost much brain, but recovered from the immediate effects of the injury, and for a good part of the time that he lived afterwards, evinced a remarkable increase of mental activity, particularly in the faculty of wit. Finally, however, disease of the brain came on and he died an idiot. A case is on record of a woman whose mind was signally improved, for a time, by an attack of typhus fever; but in this, too, the exalted tone of the brain was ultimately lost, and the woman became idiotic. It is related of the celebrated botanist, De Candolle, that in his seventh year he experienced an attack of acute hydrocephalus, from which affection, if children recover, it is well known they are apt to escape with impaired mental powers. It may be a question whether the tone of his brain was not permanently exalted by the disease.

A case in which the mind was improved by disease came not long since under our own observation. In the summer of 1847, when on a visit to the Paroquet Springs, we were applied to by Mr. William Collins, aged 61 years, for advice in his case. He was unmarried, had been a man of laborious habits, and a free liver. At this time he was laboring under extreme hypochondria, with symptoms of subacute gastritis. The least portion of animal food or ardent spirits aggravated all his distressing symptoms. For the depression of spirits he had been advised to use bitters in brandy, but was sensible that he was injured by the prescription. He was fully conscious of the disordered state of his mind, and inquired whether we had remarked anything about him unnatural. We recommended dieting, total abstinence from all stimulating drinks, and constant occupation.

The effect of the change in his habits was immediately manifest. With the subsidence of the dyspeptic symptoms he was relieved of the mental despondency under which he had labored, and rapidly regained his flesh and strength. It is more than eighteen months since he began to improve, and he now enjoys the most excellent health, with an activity of mind quite surprising to his friends. He is cheerful, extremely fond of books, and without ever having composed a line of verse previous to his illness, has become a great rhymer. We saw him a short time since and were assured by him that he learns with greater facility than he did when a boy. All his life, he informs us, he has been disposed to wakefulness, and now he sleeps only four hours in the twenty-four.

It is evident that the tone of our old patient's brain has been improved by disease, and that it is still the seat of preternatural excitement. There is danger that this may give way to changes that would bring on an opposite state of mind, or that it may be so exalted as to induce madness. The last danger would be greater, if in his habits he had not become strictly temperate.

